# Comparison of Single-Dose Cardioplegia in Valvular Heart Surgery: Lactated Ringer’s-Based del Nido *vs.* Histidine-Tryptophan-Ketoglutarate Cardioplegia Solution

**DOI:** 10.21470/1678-9741-2022-0447

**Published:** 2023-09-19

**Authors:** Narongrit Kantathut, Kasisak Luangpatom-aram, Siam Khajarern, Parinya Leelayana, Piya Cherntanomwong

**Affiliations:** 1 Department of Surgery, Division of Thoracic and Cardiovascular Surgery, Ramathibodi Hospital, Mahidol University, Bangkok, Thailand

**Keywords:** Ringer’s Solution, Valvular Surgery, Stroke Volume, Left Ventricular Function, Cardiac Arrhythmias

## Abstract

**Introduction:**

This study evaluated myocardial protection and clinical outcomes when using lactated Ringer’s solution as the base solution for del Nido cardioplegia compared with histidine-tryptophan-ketoglutarate (HTK) solution in valvular surgery.

**Methods:**

From January 2017 to May 2018, 71 adult patients who underwent valvular surgery with del Nido cardioplegia (n=37) or HTK cardioplegia (n=34) were retrospectively analyzed.

**Results:**

Patients’ characteristics were comparable between groups. Postoperative peak troponin T levels were similar. The del Nido group had a decreased incidence of ventricular fibrillation after aortic cross-clamp removal (13.51 *vs.* 55.88%; *P*<0.001), lower total volume of cardioplegia administered (1,000 [1,000, 1,250] *vs.* 1,800 [1,500, 2,000] mL; *P*<0.001), shorter hospital stay (6 [5, 8] *vs.* 7 [6, 10] days; *P*=0.03), and less postoperative red cell transfusion (34.29 *vs.* 61.11%; *P*=0.024). There is no difference in aortic cross-clamping time, postoperative change in left ventricular ejection fraction, intensive care unit stay, duration of inotropic support, new onset of atrial fibrillation, in-hospital mortality, complications, and three-year overall survival rate.

**Conclusion:**

Lactated Ringer’s-based del Nido cardioplegia can be safely used for valvular surgery with acceptable clinical outcomes compared to HTK cardioplegia.

## INTRODUCTION

Cardioplegia is one of the critical components of myocardial protection during cardiac surgery. Traditional cardioplegia is usually administered repetitively every 20 minutes. On the other hand, single-dose cardioplegia is typically administered once or with extended dosing intervals, allowing for fewer disruptions and improving the surgical workflow, which can lead to shorter aortic occlusion periods or reduced operative time^[1,2]^. Recent studies have demonstrated its usefulness and safety for myocardial protection in single cardiac valve surgery. In addition, theoretical advantages and outcome studies have led to the increased popularity of single-dose cardioplegia in complex valve procedures and minimally invasive valve surgery^[3,4,5,6]^.

Histidine-tryptophan-ketoglutarate (HTK) solution (HTK or Custodiol®; Koehler Chemi, Alsbach-Haenlien, Germany) and del Nido cardioplegia are single-dose cardioplegia available in many cardiac centers. HTK cardioplegia was first described by Bretschneider^[7]^. It provides equivalent clinical outcomes compared to traditional multidose cardioplegia^[5,8]^. In addition, it is also used in organ preservation (heart, kidney, and liver) for transplantation. At our institution, HTK cardioplegia has been the standard single-dose cardioplegia for myocardial protection in both pediatric and adult cardiac surgery for a decade.

Del Nido cardioplegia has been used exclusively in pediatric cardiac centers for myocardial protection during cardiac surgery^[9]^. Recently, del Nido cardioplegia has become more commonly used in adult cardiac surgery. Since Plasma-Lyte™ A (Baxter Healthcare Corporation, Deerfield, Illinois, United States of America), the base solution for del Nido cardioplegia, is unavailable in many countries, our institution has utilized lactated Ringer’s solution as a base solution for del Nido cardioplegia with excellent results compared to our standard blood cardioplegia^[10,11]^. To evaluate the safety and efficacy of this modified del Nido cardioplegia, we compared the clinical outcomes associated with modified del Nido cardioplegia and HTK cardioplegia in valvular heart surgery.

## METHODS

### Patients

Patients aged 18 years or older who had elective cardiac surgery for valvular heart disease using single-dose cardioplegia, either HTK or del Nido cardioplegia, were eligible for inclusion in the study. The cardiac procedure was performed through standard median sternotomy, including isolated mitral valve surgery, isolated aortic valve surgery, and multiple valve surgery. The exclusion criteria were emergency surgery, concomitant coronary bypass surgery, concomitant aortic surgery, and concomitant maze procedure. From January 2017 to May 2018, a total of 71 patients (37 in the del Nido group and 34 in the HTK group) were retrospectively reviewed. All patients were followed up until June 2021.

The primary outcome was the highest postoperative troponin T level within 24 hours. Immediately postoperative, 12-hour postoperative, and 24-hour postoperative samples were routinely obtained.

Secondary outcomes were postoperative outcomes, intraoperative outcomes, and assessments of additional measures of impaired myocardial protection. Postoperative outcomes consisted of the intensive care unit (ICU) stay, hospital stay, mortality, occurrence of postoperative complications, and the need for postoperative red cell transfusion. Intraoperative outcomes included the total volume of cardioplegia administered, the number of doses, total cardiopulmonary bypass (CPB) time, and aortic cross-clamping time. Assessments of additional measures of impaired myocardial protection included the incidence of ventricular fibrillation after aortic cross-clamp removal, postoperative changes in left ventricular ejection fraction (LVEF), duration of inotrope/ vasopressor requirement, incidence of new-onset postoperative atrial fibrillation or flutter, and requirement for intra-aortic balloon pump (IABP) support.

Before and after the procedure, LVEF was evaluated by intraoperative transesophageal echocardiography. Postoperative changes in LVEF were defined as the difference between postoperative LVEF and preoperative LVEF. Patient characteristics variables and postoperative outcomes (including Society of Thoracic Surgeons [STS] risk score, mortality, renal failure, prolonged ventilation, stroke, deep sternal wound infection, and reoperation) were as defined by the STS Adult Cardiac Surgery Database.

### Cardioplegia and Delivery

The compositions of HTK and del Nido cardioplegias are described in Table 1. Our modified del Nido cardioplegia was sterilely prepared by an in-house pharmacist. It was preserved refrigerated at a temperature of 2-8°C and used within 24 hours. On delivery, it was mixed 1:4 with one part of oxygenated pump blood to four parts of cardioplegia solution. The cardioplegia in our CPB circuit travels through a coil heat exchanger-equipped, non-recirculating cardioplegia set. Delivery temperature was at 4°C. The method of delivery is determined by the degree of aortic valve insufficiency. Del Nido cardioplegia was usually administered antegradely through an aortic root catheter. If the patients had severe aortic valve insufficiency, the cardioplegia was given directly through the coronary ostia. Our cardioplegia strategy was to infuse a single dose of 20 mL/kg with a maximum dose of 1,000 mL for patients weighing > 50 kg over 1-2 minutes with a system pressure of 100-200 mmHg. After 90 minutes of aortic cross-clamping time, the need for redosing and the amount of subsequent dose depended on the surgeon’s decision^[1,9,10,11]^.

In patients receiving HTK cardioplegia, approximately 1,500-2,000 mL of HTK solution was infused with hydrostatic pressure (from approximately 2 m height) over 6-8 minutes through an aortic root catheter or directly through the coronary ostia depending on the degree of aortic valve insufficiency. Generally, redosing was not required unless there was electrical activity. Additional doses of 100-200 mL were administered as needed. HTK solution was delivered at a temperature of 4-8°C.

All patients received ultrafiltration during the operation.

### Ethical Statement

The study protocol was approved by the institutional review board of Ramathibodi Hospital (ref No. 086027, date of approval: 09/06/2017), with patient consent waived.

### Statistical Analyses

The sample size for the primary outcome was calculated by the difference between 24-hour postoperative troponin levels of the HTK and del Nido cardioplegia groups reported by Talwar et al.^[12]^. To detect the statistical difference in a two-sided test with 5% alpha error and 90% power, 26 patients were required in each group.

**Table 1:** Cardioplegia composition.

Modified del Nido cardioplegia		Custodiol®-HTK	
Lactated Ringer’s solution	1000 mL	Sodium chloride	15 mmol/L
Sodium bicarbonate 1 mEq/mL	13 mL	Potassium chloride	9 mmol/L
Mannitol (20%)	16.3 mL	Magnesium chloride	4 mmol/L
Magnesium sulfate (50%)	4 mL	Calcium chloride	0.015 mmol/L
Lidocaine (1%)	13 mL	Histidine	198 mmol/L
Potassium chloride 2 mEq/mL	13 mL	Tryptophan	2 mmol/L
		Ketoglutarate	1 mmol/L
		Mannitol	30 mmol/L
Dose: 20 mL/kg with a maximum dose of 1,000 mL for patients weighing > 50 kg		Dose: 1,500 – 2,000 mL	

HTK=histidine-tryptophan-ketoglutarate

Continuous variables were expressed as mean (standard deviation) or median (interquartile range) and were compared using an independent sample *t*-test or the Mann-Whitney U test. Categorical variables were expressed as frequencies and percentages and were analyzed using chi-squared or Fisher’s exact test. Survival analyses were estimated by the Kaplan-Meier method. STATA version 14 (College Station, Texas, United States of America) was used for statistical analysis. A *P*-value < 0.05 is considered a statistical significance.

## RESULTS

### Patients’ Characteristics

Most of the patients’ characteristics were similar between groups, except patients with dyslipidemia were higher in the del Nido group (43.24 *vs.* 20.59 %, *P*=0.042), and preoperative LVEF was lower in the del Nido group (60 [50, 63] *vs.* 66 [60, 69] %, *P*=0.002) than in the HTK group (Table 2).

**Table 2:** Patients’ characteristics.

Variables	del Nido (n = 37)	HTK (n = 34)	*P*-value
Age, years, median (IQR)	60 (52, 67)	58 (52, 62)	0.632
Gender, n (%)			
Male	19 (51.35)	14 (41.18)	0.390
Female	18 (48.65)	20 (58.82)	
BSA (m^2^), mean (± SD)	1.65 (± 0.19)	1.64 (± 0.16)	0.771
STS risk score (%), median (IQR)	1.026 (0.647, 1.831)	1.042 (0.546, 1.731)	0.632
Preoperative LVEF (%), median (IQR)	60 (50, 63)	66 (60, 69)	0.002
Comorbidities, n (%)			
Diabetes	7 (18.92)	7 (20.59)	0.860
Hypertension	22 (59.46)	19 (55.88)	0.761
Dyslipidemia	16 (43.24)	7 (20.59)	0.042
Dialysis	1 (2.70)	2 (5.88)	0.604
Chronic kidney disease	4 (10.81)	2 (5.88)	0.675
Cerebrovascular disease	5 (13.51)	3 (8.82)	0.712
Atrial fibrillation	11 (29.73)	10 (29.41)	0.977
NYHA, n (%)			
Class I	2 (5.41)	6 (17.65)	0.120
Class II	28 (75.68)	25 (73.53)	
Class III	7 (18.92)	2 (5.88)	
Class IV	0	1 (2.94)	
Operation, n (%)			
Isolated valve surgery			
Mitral	14 (37.84)	20 (58.82)	0.077
Aortic	11 (29.73)	4 (11.76)	0.064
Combine valve surgery			
Aortic + mitral	4 (10.81)	3 (8.82)	0.779
Mitral + tricuspid	5 (13.51)	7 (20.59)	0.427
Aortic + tricuspid	1 (2.70)	0	0.334
Aortic + mitral + tricuspid	2 (5.41)	0	0.169

BSA=body surface area; HTK=histidine-tryptophan-ketoglutarate; IQR=interquartile range; LVEF=left ventricular ejection fraction; NYHA=New York Heart Association; SD=standard deviation; STS=Society of Thoracic Surgeons

### Primary Outcomes

**Fig. 1:**
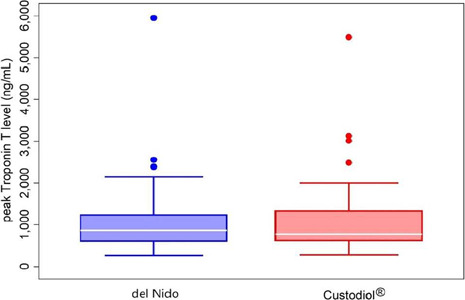
The highest postoperative troponin T level of the del Nido and histidine-tryptophan-ketoglutarate groups.

The highest postoperative troponin T level within 24 hours was similar between the del Nido and HTK groups (0.875 [0.611, 1.235] *vs.* 0.785 [0.622, 1.334] ng/mL, *P*=0.687) (Figure 1).

### Secondary Outcomes: Intraoperative Outcomes

The del Nido group had a decreased incidence of ventricular fibrillation after aortic cross-clamp removal (13.51 *vs.* 55.88%, *P*<0.001) compared with the HTK group. Postoperative changes in LVEF were similar (0 [-2, 1] *vs.* 0 [-8, 0] %, *P*=0.24) between the groups. The total cardioplegia volume in the del Nido group was significantly lower than that in the HTK group (1,000 [1,000, 1,250] *vs.* 1,800 [1,500, 2,000] mL, *P*<0.001). The number of doses, the aortic cross-clamping time, and total bypass time were similar between groups (Table 3).

**Table 3:** Intraoperative and postoperative outcomes.

Variables	del Nido (n = 37)	HTK (n = 34)	*P*-value
Intraoperative			
Total cardioplegia volume (mL), median (IQR)	1,000 (1,000, 1250)	1,800 (1,500, 2,000)	< 0.001
Number of doses, mean (± SD)	1 (1, 2)	1 (1, 2)	0.806
Cross-clamping time (minutes), mean (± SD)	105.72 (± 36.18)	104.85 (± 26.68)	0.909
Total CPB time (minutes), mean (± SD)	139.70 (± 44.89)	145.85 (± 39.68)	0.544
Ventricular fibrillation after aortic cross-clamp removal, n (%)	5 (13.51)	19 (55.88)	< 0.001
Postoperative LVEF (%), median (IQR)	60 (50, 63)	61 (59, 67)	0.073
LVEF change (%), median (IQR)	0 (-2, 1)	0 (-8, 0)	0.240
Postoperative			
ICU stay (days), median (IQR)	2 (2, 3)	3 (2, 4)	0.120
Hospital stay (days), median (IQR)	6 (5, 8)	7 (6, 10)	0.030
Inotrope/vasopressor requirement (days), median (IQR)	1 (1, 1)	1 (1, 2)	0.214
Postoperative atrial fibrillation or flutter, n (%)	2 (7.69)	5 (20.83)	0.239
Complication, n (%)	6 (16.22)	8 (23.53)	0.439
Renal failure, n (%)	1 (2.70)	0	0.999
Prolonged ventilation > 24 hours, n (%)	2 (5.41	5 (14.71)	0.248
Stroke, n (%)	1 (2.70)	0	0.999
Reoperation, n (%)	2 (5.41)	3 (8.82)	0.574
Deep sternal wound infection, n (%)	0	0	-
Hospital death, n (%)	0	0	-
IABP, n (%)	2 (5.41)	3 (8.82)	0.574
Red cell transfusion, n (%)	14 (37.84)	22 (64.71)	0.024

CPB=cardiopulmonary bypass; HTK=histidine-tryptophan-ketoglutarate; IABP=intra-aortic balloon pump; IQR=interquartile range; LVEF=left ventricular ejection fraction; SD=standard deviation

### Postoperative Outcomes

No in-hospital mortality occurred. Postoperative complications, duration of inotrope/vasopressor requirement, the incidence of new-onset postoperative atrial fibrillation or flutter, the requirement for IABP support, and the ICU stay were similar between the groups. The del Nido group had a lower incidence of postoperative red cell transfusion (37.84 *vs.* 64.71%, *P*=0.024) and a shorter hospital stay (6 [5, 8] *vs.* 7 [6, 10] days, *P*=0.03) than the HTK group (Table 3).

### Survival Analyses

**Fig. 2:**
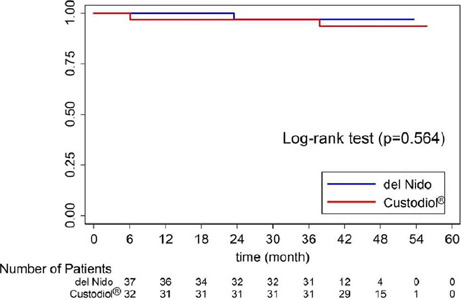
Rate of survival in del Nido cardioplegia (blue) and histidine-tryptophan-ketoglutarate cardioplegia (red) groups.

The median follow-up time was 43.3 (38, 48.2) months. Three deaths occurred during the follow-up period (one in the del Nido group and two in the HTK group). At 36 months, the cumulative survival rate was 96.97% and 96.88% for the del Nido and the HTK group, respectively. The Kaplan-Meier survival analysis of the del Nido group *vs.* the HTK group found no significant difference between survival distributions (log-rank *P*=0.564) (Figure 2).

## DISCUSSION

This present study on adult valvular surgery suggested equivalent myocardial protection and clinical outcomes with lactated Ringer’s-based del Nido cardioplegia as compared to HTK cardioplegia. Assessment of myocardial injury, such as peak troponin T levels, the incidence of new-onset postoperative atrial fibrillation or flutter, duration of inotrope/vasopressor support, the requirement for IABP support, and postoperative change in LVEF were similar between the cardioplegia protocols. Notably, the incidence of ventricular fibrillation after aortic cross-clamp removal was lower in del Nido cardioplegia. Additionally, the use of del Nido cardioplegia was associated with a lower total volume of cardioplegia, shorter length of hospital stay, and lower incidence of postoperative red cell transfusion.

Administration of cardioplegia during the exposure of the valvular structure in valvular heart surgery is challenging. In mitral valve surgery, over-traction of the mitral retractor can distort the aortic valve. Therefore, the mitral retractor must be periodically dismounted during the administration of antegrade cardioplegia to ensure aortic valve competency and appropriate delivery to the coronary arteries. In aortic valve surgery, the cardioplegia must be delivered through the coronary ostia. Repetitive manipulation around the coronary ostia may result in a coronary ostial injury. In addition, cardioplegia distribution may not be sufficient from the unbalanced coronary ostial infusion. These maneuvers usually interrupt the surgical workflow when used in conjunction with traditional multidose cardioplegia. The use of retrograde cardioplegia may be a suitable option in this situation. However, it is associated with insufficient protection of the right ventricle^[13]^. Malposition and dislodging of the coronary sinus catheter can occur during cardiac procedure and lead to undesirable complications such as rupture of the coronary sinus. Moreover, retrograde cardioplegia could not always be achieved, especially in a minimally invasive approach. Thus, a single-dose cardioplegia is an attractive option for myocardial protection during valvular heart surgery. Its longer redosing interval provides an uninterrupted, streamlined surgical workflow. Additionally, fewer manipulations potentially reduce the risk of serious complications.

Myocardial injury, represented by peak postoperative troponin T levels in this study, was similar between cardioplegia protocols. Both del Nido and HTK cardioplegias have been reported to have similar or superior myocardial protection compared to the blood cardioplegia strategy^[1,2,3,5,8,11]^. However, very few studies have a face-to-face comparison between the two cardioplegia protocols. In pediatric patients who underwent elective surgical correction for tetralogy of Fallot, the randomized study by Talwar et al.^[12]^ demonstrated lower postoperative troponin release in the del Nido group than in those who received HTK solution. Lee et al.^[6]^ also reported similar results in minimally invasive cardiac surgery patients. The generally acceptable time limit for a singledose infusion of del Nido cardioplegia is 60-90 minutes^[1,3,9,10,11,14]^. However, if necessary, additional doses can be administered after 90 minutes of the initial dose. In comparison, the additional dose of HTK cardioplegia may not be required within 180 minutes of the initial dose unless there is a spontaneous return of cardiac electrical activity. Most of these previous studies were performed in low-risk, non-complex, single-valve patients with preserved ventricular function. Therefore, the aortic cross-clamping time is usually < 90 minutes. In our study, the mean aortic cross-clamping time in the del Nido and HTK groups was longer than in other studies (an average of 105 and 104 minutes, respectively) which could be explained by a more complex procedure (*e.g.*, multiple valves surgery) included in our study. Our study’s prolonged aortic cross-clamping time could have been sufficient to produce higher troponin release postoperatively in both groups. Therefore, there was no difference in postoperative troponin levels. Similar results for prolonged aortic-cross clamping time were also observed in Duan et al.^[4]^ complex valve surgery study.

Ventricular fibrillation can occur when the heart muscle is damaged or stressed. This can happen when the heart is exposed to ischemia, hypoxia, or other forms of stress. Therefore, the occurrence of ventricular fibrillation may be an indicator of poor myocardial protection during surgical procedures. According to the finding from Elcik et al.^[15]^, at the messenger ribonucleic acid (mRNA) expression level of associated markers, HTK cardioplegia caused more damage to the myocardium compared to blood cardioplegia. In addition, a significant increase in ventricular fibrillation after aortic cross-clamp removal with HTK cardioplegia has been consistently reported^[4,12]^. Our study also demonstrated a higher incidence of ventricular fibrillation in the HTK group compared to the del Nido group. This finding may indirectly indicate better myocardial protection with del Nido cardioplegia. However, these findings had no impact on outcomes, as demonstrated by similar postoperative troponin levels and clinical outcomes. On the other hand, another interesting finding is that the use of del Nido cardioplegia is associated with a lower incidence of ventricular fibrillation after aortic cross-clamp removal^[2,4,11,12,14]^. One of the ingredients of del Nido cardioplegia, lidocaine, may be responsible for these findings.

The total volume of HTK cardioplegia is generally higher than of del Nido cardioplegia. Additionally, HTK is crystalloid cardioplegia, whereas del Nido cardioplegia is 1:4 blood cardioplegia. Therefore, more hemodilution may occur in patients who receive HTK cardioplegia. Many del Nido cardioplegia studies have reported a reduction in the total cardioplegia volume. The reduction in the total volume of cardioplegia may result in less hemodilution and a lower incidence of postoperative red cell transfusion^[2,3,4,11,12]^. In our study, del Nido cardioplegia was also associated with a lower total volume of cardioplegia and a lower incidence of postoperative red cell transfusion. Although the perfused solution can be removed from the coronary sinus to reduce hemodilution, these unnecessary procedures added on to the operation, including additional venous cannulation, total caval occlusion, and right atrial incision, can prolong the operation and lead to undesirable outcomes.

Plasma-Lyte™, the base solution of original del Nido cardioplegia, has no calcium. The final calcium concentration of the delivered cardioplegia can be considered as trace after mixing one part of oxygenated pump blood with the crystalloid component^[9]^. In contrast, lactated Ringer’s solution has calcium in a range of 1.5-3 mEq/L. Our modification to utilize lactated Ringer’s as a based solution leads to higher calcium concentration in the delivered cardioplegia^[11]^. Theoretically, this may increase intracellular calcium accumulation, resulting in poor myocardial recovery. Although the calcium concentration in this modified version of del Nido cardioplegia was a concern, there was no evidence of impaired outcomes with our cardioplegia strategy in both short-term and midterm follow-ups.

### Limitations

This study was limited by its retrospective, non-randomized, and single-institution design. We included various procedure types in this investigation, although the proportion of operation types was not statistically different. In addition, we could not control confounding variables due to the small sample size. As the goal of this study was to compare our modified del Nido cardioplegia with HTK cardioplegia, the findings in this single-institution study may not be generalizable or applicable to other cardiac centers because the variations in cardioplegia protocol may not provide comparable results. To validate our findings, a larger sample size or randomized controlled study to compare our modified del Nido cardioplegia and HTK cardioplegia is required.

## CONCLUSION

The use of lactated Ringer’s-based del Nido cardioplegia is a safe and effective alternative to HTK cardioplegia for myocardial protection in valvular surgery. Our findings demonstrated that the modified del Nido cardioplegia provided comparable outcomes with several advantages, including a decreased incidence of ventricular fibrillation after aortic cross-clamp removal, lower total cardioplegia volume, shorter length of hospital stay, and a lower incidence of postoperative red cell transfusion. However, further investigation in a large-scale or randomized controlled study is warranted to confirm our findings.

## Abbreviations, Acronyms & Symbols

BSA: Body surface area
CPB: Cardiopulmonary bypass
HTK: Histidine-tryptophan-ketoglutarate
IABP: Intra-aortic balloon pump
ICU: Intensive care unit
IQR: Interquartile range
LVEF: Left ventricular ejection fraction
NYHA: New York Heart Association
SD: Standard deviation
STS: Society of Thoracic Surgeons
